# Cloning and Expression of *Col10a1* Gene and Its Response to Wnt/TGF-β Signaling Inhibitors in the Chinese Three-Keeled Pond Turtle (*Mauremys reevesii*)

**DOI:** 10.3390/ani15223315

**Published:** 2025-11-17

**Authors:** Yue Li, Junxian Zhu, Tong Ren, Xiaoli Liu, Chen Chen, Liqin Ji, Xiaoyou Hong, Chengqing Wei, Haigang Chen, Xinping Zhu, Wei Li, Lihong Dang

**Affiliations:** 1 School of Biological Sciences and Engineering, Shaanxi University of Technology, Hanzhong 723001, China; liyue20001215@163.com; 2 Key Laboratory of Tropical & Subtropical Fishery Resource Application & Cultivation of Ministry of Agriculture and Rural Affairs, Pearl River Fisheries Research Institute, Chinese Academy of Fishery Sciences, Guangzhou 510380, China; zhujunxian_1994@163.com (J.Z.); liu_xiaoli1988@126.com (X.L.); chenchen3729@163.com (C.C.); jiliqin@prfri.ac.cn (L.J.); hongxiaoyou1216@163.com (X.H.); wcq1970@163.com (C.W.); harbourchen66@163.com (H.C.); zhuxinping_1964@163.com (X.Z.); 3 South China Sea Fisheries Research Institute, Chinese Academy of Fishery Sciences, Guangzhou 510300, China; 4 Science and Technology Research Center of China Customs, Beijing 100026, China; blue2603@163.com

**Keywords:** *Mauremys reevesii*, *Col10a1*, carapace, collagen, endochondral ossification

## Abstract

The formation of the Chinese three-keeled pond turtle (*Mauremys reevesii*) carapace is a complex biological event involving developmental processes such as collagen deposition and ossification. As a key regulator of collagen deposition and ossification, *Col10a1* may play a crucial role in carapace development, though its specific mechanism remains unclear. To investigate the role of *Col10a1* during carapace development and its regulatory mechanisms, we cloned its cDNA sequence and performed bioinformatic analysis. This revealed that *Col10a1* encodes a stable, hydrophilic, and basic protein, with phylogenetic analysis showing closest evolutionary relationships to other reptiles and the greatest divergence from fish. Further RT-qPCR analysis examined *Col10a1* expression patterns in *M. reevesii* embryos at stages 14, 18, and 22, as well as in various tissues of adult males and females. Results indicated that during embryonic development, *Col10a1* expression levels progressively increased alongside the progression of carapace ossification and collagen deposition, suggesting its involvement in regulating this process. In adult tissues, *Col10a1* exhibited widespread expression, with particularly high levels in the brain, kidneys, and liver, suggesting potential specialized functions in these organs. Finally, in vitro experiments demonstrated that inhibition of the Wnt/β-catenin pathway with salinomycin sodium salt downregulated the expression of both its target genes (*Sp5*, *Myc*, *Ccnd1*) and *Col10a1*. In contrast, inhibition of the TGF-β/Smad pathway with oxymatrine suppressed its target genes (*Serpine1*, *Cdkn1a*) but concomitantly upregulated *Col10a1*. These results suggest that *Col10a1* expression may be positively regulated by the Wnt/β-catenin pathway and negatively regulated by the TGF-β/Smad pathway. Our findings provide novel insights into the molecular mechanisms governing carapace development and collagen deposition in *M. reevesii*, laying a crucial foundation for further investigations into the regulatory networks involving *Col10a1* during carapace formation.

## Simple Summary

The formation of *Mauremys reevesii* carapaces requires precisely regulated collagen deposition and ossification. The *Col10a1* gene may play a key role in this process, but its specific function remains unclear. In this study, we aim to investigate the role of *Col10a1* in *M. reevesii* carapace formation through molecular cloning, expression profiling, and functional characterization. We successfully cloned the *Col10a1* gene and characterized its molecular properties using bioinformatics tools. Furthermore, we sought through spatiotemporal expression analysis at key embryonic developmental stages and in multiple adult tissues, combined with in vitro experiments using *M. reevesii* carapace-derived cells, to investigate the potential regulatory mechanisms of the *Col10a1*. Our findings provide important insights into the molecular mechanisms underlying *M. reevesii* carapace development and lay a foundation for further investigations into reptilian carapace formation mechanisms.

## 1. Introduction

As a key structural component of the extracellular matrix (ECM), collagen is the most abundant and widely distributed functional protein in animals [1,2]. It plays crucial roles in maintaining the mechanical properties and physiological functions of various tissues [3]. At present, mammalian tissues are the primary sources for collagen extraction [4]. However, their use is limited by zoonotic disease safety concerns and regional religious restrictions [1,4,5], driving the search for safer alternatives. Among these, aquatic collagen has garnered increased attention, with sources including fish [6], starfish [7], jellyfish [8], and turtle/tortoise [9]. In particular, turtle-and-tortoise-derived collagen has emerged as a high-quality alternative with considerable potential. The Chinese three-keeled pond turtle (*Mauremys reevesii*) is a commercially important aquaculture species in China, valued for culinary and medicinal purposes [10,11]. Its carapace, a traditional Chinese medicinal ingredient rich in collagen and other bioactive compounds [12], is a key raw material for producing turtle shell gelatin, turtle shell peptides, and the traditional Chinese medicinal dessert turtle jelly [13,14]. However, the mechanisms of collagen biosynthesis and deposition in the *M. reevesii* carapace remain poorly investigated.

The *Col10a1* is a pivotal member of the collagen gene family, playing an essential role in collagen biosynthesis [15]. It encodes the alpha-1 chain of type X collagen, a short-chain secreted protein initially identified in the tibial growth plate cartilage of chicken embryos [16,17]. As a specific marker of the ECM in hypertrophic chondrocytes, *Col10a1* is integral to endochondral ossification, where it facilitates matrix mineralization and compartmentalization of matrix components, thereby critically supporting the ossification process within skeletal growth plate cartilage [18,19]. Furthermore, *Col10a1* mediates the remodeling of the growth plate cartilage ECM, constructing a microenvironment conducive to angiogenesis and bone replacement. It forms a directed scaffold on the surface of degraded cartilage templates, thereby promoting the directed migration of osteoprogenitor cells and the ordered deposition of type I collagen [20,21]. The role of *Col10a1* has been primarily characterized in mammals and birds, whereas its function in reptiles—particularly in unique skeletal structures like the turtle carapace—remains poorly understood. This knowledge gap hinders a comprehensive understanding of both its evolutionary conservation and its potential lineage-specific functions in reptilian skeletal development. Previous studies from our group revealed significant upregulation of *Col10a1* during *M. reevesii* carapace collagen synthesis, suggesting its potential involvement in carapace collagen matrix construction.

Collagen metabolism and skeletal development are closely linked to the Wnt/β-catenin and TGF-β/Smad signaling pathways. The Wnt/β-catenin pathway regulates the differentiation of chondrocyte precursors into hypertrophic chondrocytes by maintaining their proliferative state, thereby providing the cellular foundation for subsequent endochondral ossification [22]. Concurrently, the TGF-β/Smad signaling pathway contributes to early cartilage template establishment by promoting mesenchymal cell aggregation and cartilage nodule formation. It also influences the stability and remodeling of the collagen fiber network by regulating the dynamic balance between matrix metalloproteinases (MMPs) and their inhibitors (TIMPs) [23]. Thus, the spatiotemporal coordination of these two pathways during *M. reevesii* carapace formation may provide a unique regulatory model for *Col10a1*-mediated collagen deposition and endochondral ossification. However, systematic investigations into the expression characteristics and potential biological functions of *Col10a1* in *M. reevesii* carapace collagen formation remain scarce. To address this knowledge gap, the present study cloned the cDNA sequence of *Col10a1* from *M. reevesii*, performed bioinformatics analysis on its sequence, explored its spatial-temporal expression patterns in carapace tissues at different embryonic stages and adult male/female tissues, and investigated the gene’s response to Wnt/β-catenin and TGF-β/Smad inhibitor treatments. These findings will provide foundational data for further investigation into the molecular mechanisms of *Col10a1* in *M. reevesii* carapace collagen deposition and endochondral ossification.

## 2. Materials and Methods

### 2.1. Experimental Animals and Ethical Declaration

The *M. reevesii* individuals used in this experiment were three-winter-old adults obtained from the Turtle and Soft-shelled Turtle Breeding and Conservation Base of the Pearl River Fisheries Research Institute, Chinese Academy of Fishery Sciences (Guangzhou, China). A total of six healthy individuals were selected, comprising three females and three males. Following anesthesia and blood collection, tissues including liver, kidney, spleen, heart, lung, muscle, brain, testis, and ovaries were harvested. These tissues were placed in enzyme-free cryopreservation tubes and snap-frozen in liquid nitrogen.

Additionally, 200 fertilized eggs of *M. reevesii* were collected. The eggs were buried in vermiculite (vermiculite-to-water ratio, 1:1) with the fertilization spots facing upward, and incubated in a constant-temperature incubator (Tai Hong Zhu Jiang, model FHX-250, 250 L) maintained at 32 ± 1 °C [24]. Embryonic developmental stages of *M. reevesii* were primarily determined according to the morphological criteria established by Tokita and Kuratani [25], supplemented by the criteria proposed by Greenbaum [26]. Three fertilized eggs were dissected daily for developmental stage identification. At stage 14 (formation of the primitive carapace margin), stage 18 (appearance of pigment deposition and keel initiation), and stage 22 (fully developed morphology with defined structures), carapace samples were collected from 30 fertilized eggs per stage. Freshly collected carapace samples were rapidly frozen in liquid nitrogen and stored at −80 °C.

All animal experiments were conducted in accordance with protocols approved by the Laboratory Animal Ethics Committee of the Pearl River Fisheries Research Institute, Chinese Academy of Fishery Sciences.

### 2.2. Total RNA Extraction and cDNA Preparation

Total RNA was isolated from various adult *M. reevesii* tissues and from embryonic carapaces at stages 14, 18, and 22 using Trizol reagent (Ambion, Austin, TX, USA). RNA concentration was measured using the NanoQTM Nucleic Acid Detector (Thermo Scientific, Madison, WI, USA), and its integrity was assessed by 1% agarose gel electrophoresis. Subsequently, the first-strand cDNA was synthesized with the HiScript^®^ III RT SuperMix for qPCR (+gDNA wiper) reverse transcription kit (Vazyme, Nanjing, China) and stored at −20 °C for later use.

### 2.3. Cloning of the Col10a1 Gene

Gene-specific primers (Table 1) were designed based on the *M. reevesii Col10a1* cDNA sequence (GenBank accession no. XM_039529706.1) and synthesized by Guangzhou Tianyi Huiyuan Gene Technology Co., Ltd. (Guangzhou, China). Primer specificity, verified by NCBI BLAST, was further confirmed by single-peak melting curves (Supplementary Figure S1). The qRT-PCR standard curves showed high linearity (R^2^ ≥ 0.99), with slopes ranging from −3.45 to −3.21, corresponding to amplification efficiencies of 94% to 104% (Supplementary Figure S2). Polymerase chain reaction (PCR) was performed using *M. reevesii* carapace-derived cDNA to amplify the coding sequence (CDS) of *Col10a1* with the specific primer pair *Col10a1*-F/*Col10a1*-R (Table 1). The reaction system consisted of a 20 μL volume: 10 μL 2× SanTaq PCR Master Mix, 7 μL ddH_2_O, 1.0 μL cDNA template, and 1.0 μL each of Col10a1-F and Col10a1-R primers. Amplification program: 95 °C pre-denaturation for 5 min; 35 cycles of 95 °C for 30 s, 58 °C for 30 s, 72 °C for 2 min; 72 °C extension for 10 min. Amplified products were analyzed by 1% agarose gel electrophoresis, and the target band was excised and purified using a Gel Extraction Kit (Omega, Norcross, GA, USA). Subsequently, the purified product was ligated into the pMD19-T vector (TaKaRa, Beijing, China), and transformed into DH5α competent cells (Vazyme, Nanjing, China). Transformed cells were plated on LB agar, and single colonies were selected and screened by colony PCR. Positive clones were cultured, and plasmid DNA was submitted to Guangzhou Tianyi Huiyuan Gene Technology Co., Ltd. for Sanger sequencing.

### 2.4. Bioinformatics Analysis of the Col10a1

The cDNA sequences obtained from sequencing were translated into amino acid sequences using DNAMAN 8 [27]. Multiple sequence alignment was performed with ClustalW (BioEdit) software, and amino acid sequence homology analysis was conducted using BLAST [28]. Physicochemical properties, hydrophilicity and hydrophobicity profiles, signal peptides, and potential phosphorylation sites were predicted with ProtParam, ProtScale, SignalP-6.0, and NetPhos 3.1, respectively [29]. The secondary and tertiary structures of the Col10a1 protein were predicted using SOPMA [30] and SWISS-MODEL [31]. Protein domains were predicted using SMART (https://smart.embl.de (accessed on 30 June 2025)). A phylogenetic tree was constructed using the maximum likelihood (ML) method in MEGA 11 [32], with 1000 bootstrap replicates. Using the MEME Suite, conserved motifs within the Col10a1 protein were predicted. Domain annotation was then performed with the National Center for Biotechnology Information (NCBI) Conserved Domain Database (CDD). All sequences used for the analyses, including amino acid homology comparisons, phylogenetic construction, conserved motifs and structural domain analysis, were obtained from the NCBI. The corresponding accession numbers are provided in Table 2.

### 2.5. Expression Analysis of the Col10a1 Gene in Different Tissues of M. reevesii

The *Gapdh* gene was used as the housekeeping gene for normalization [33]. Both the reference and target gene primers for RT-qPCR are listed in Table 1. Using cDNA templates from various adult *M. reevesii* tissues and embryonic carapaces, the relative expression levels of the *Col10a1* were detected using the Applied Biosystems StepOnePlus Real-Time PCR Systems (Applied Biosystems, Singapore). The reaction system consisted of 20.0 μL: 10 μL iTaq Universal SYBR^®^ Green Supermix (Bio-Rad, Hercules, CA, USA), 7.0 μL ddH_2_O, 1.0 μL each of *Col10a1*-qF and *Col10a1*-qR (Table 1) primers, and 1.0 μL cDNA template. The qPCR conditions for all target genes were as follows: 95 °C for 5 min; 40 cycles of 95 °C for 10 s, 60 °C for 20 s, and 72 °C for 20 s. The melting curve analysis was performed at 95 °C for 15 s, 60 °C for 60 s, and 95 °C for 15 s.

### 2.6. Treatment of M. reevesii Carapace Cells with Salinomycin Sodium Salt and Oxymatrine

The *M. reevesii* carapace cells (MRCCs) line used in this experiment was previously established by our research group. This cell line was established by isolating carapace tissue during *M. reevesii* embryonic development and performing primary culture using the tissue block adhesion method, ultimately yielding a stably proliferating cell population. Well-growing MRCCs were seeded at appropriate concentrations into six-well plates. Upon reaching 60–70% confluence, salinomycin sodium salt (Wnt/β-catenin pathway inhibitor) [34] and oxymatrine (TGF-β/Smad pathway inhibitor) [35] stock solutions using DMSO according to manufacturer instructions. The prepared drug solutions were added to the cell culture system, with an equal volume of DMSO solvent added as a negative control group. After 24 h of drug treatment, total RNA was isolated from collected cell samples and reverse-transcribed into cDNA. The expression levels of *Col10a1* and key genes in the Wnt (*Sp5*, *Myc*, *Ccnd1*) and TGF-β (*Serpine1*, *Cdkn1a*) signaling pathways were quantified by RT-qPCR. Primer information is shown in Table 1.

### 2.7. Data Analysis

Data are presented as the mean of three independent biological replicates. Gene expression levels were determined using the 2^−ΔΔCt^ method [36]. For statistical comparison, one-way analysis of variance (ANOVA) coupled with least significant difference (LSD) test was applied in SPSS 21.0, and differences with *p* < 0.05 were deemed statistically significant.

## 3. Results

### 3.1. Cloning and Sequence Analysis of the M. reevesii Col10a1

The coding sequence (CDS) of the *Col10a1* gene was successfully cloned from *M. reevesii* carapace-derived cDNA. Colony PCR yielded a single band of approximately 2000 bp (Supplementary Figure S3), and sequencing confirmed a synonymous single-base mutation (T to C) compared to the NCBI reference sequence (Supplementary Figure S4).

The cloned cDNA is 2194 bp in length and contains a 2034 bp CDS encoding a protein of 677 amino acid (Supplementary Figure S5a). Bioinformatic analysis predicted the encoded protein to be alkaline and stable, with a theoretical isoelectric point (pI) of 9.39, an instability index of 25.91, and an aliphatic index of 58.26. Hydrophobicity analysis identified the most hydrophobic residue at position 8 (score: 2.744) and the most hydrophilic at position 577 (score: −3.233) (Supplementary Figure S5b). Furthermore, the *M. reevesii* Col10a1 was predicted to contain 28 phosphorylation sites and an N-terminal 18-amino-acid signal peptide (Supplementary Figure S5c,d). The secondary structure of M. reevesii Col10a1 was predicted to be predominantly composed of random coils (73.71%), along with extended strands (11.08%), β-turns (9.31%), and α-helices (5.91%) (Supplementary Figure S5e), and the predicted tertiary structure was consistent with these secondary structure elements (Supplementary Figure S5f). Domain analysis identified two low-complexity regions, five collagen-specific domains with Gly-X-Y repeats, and a conserved C-terminal globular C1q domain (NC1) (Supplementary Figure S5g) [37].

### 3.2. Amino Acid Similarity Analysis of Col10a1 Protein and Construction of Phylogenetic Tree

**Figure 1:**
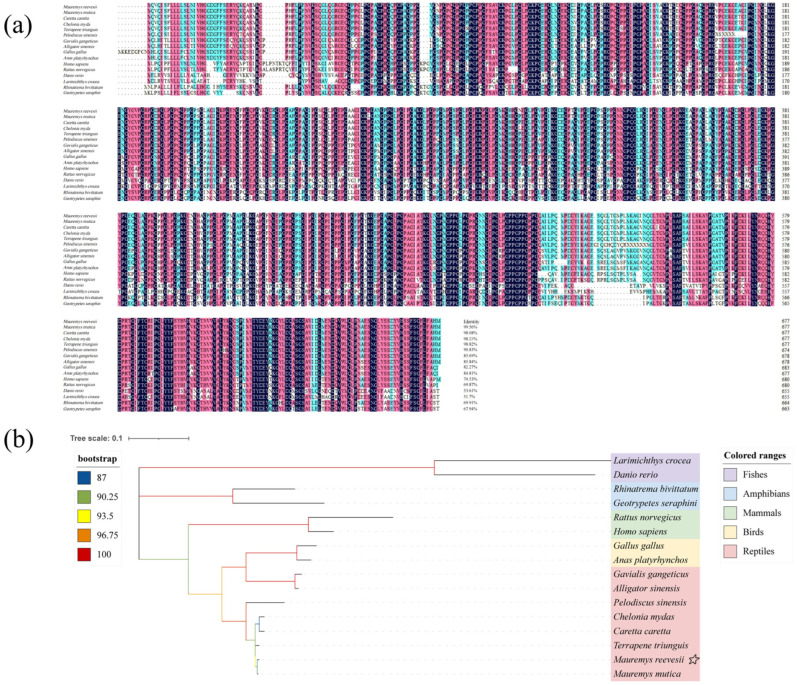
The comparison of amino acid sequence homology similarity and phylogenetic tree of Col10a1 between *M. reevesii* and other species. (**a**) amino acid sequence homology similarity; (**b**) phylogenetic tree. Notation: (**b**). The yellow star symbol indicates *M. reevesii*, the target species of this study, which is marked as a key node in this phylogenetic tree.

Amino acid sequence homology alignment results indicate that the *M. reevesii* Col10a1 exhibits high identity with reptiles, showing the highest identity with *Mauremys mutica* at 99.56%. It also demonstrates significant identity with other turtle and tortoise species, including *Caretta caretta* (98.08%), *Chelonia mydas* (98.23%), *Terrapene triunguis* (98.82%), and *Pelodiscus sinensis* (90.83%) (Figure 1a). However, the Col10a1 exhibits low conservation in fish, with only 51.70% identity with *Larimichthys crocea*. Phylogenetic tree results (Figure 1b) indicate that *M. reevesii* Col10a1 clusters with reptilian species, forming a clade most closely related to *M. mutica* and showing the greatest evolutionary distance from fish species. This finding fully aligns with amino acid sequence homology analysis, together confirming the conservation of Col10a1 across turtle and soft-shelled turtle species.

### 3.3. Conserved Motifs and Structural Domain Analysis of Col10a1 Proteins Across Different Species

**Figure 2:**
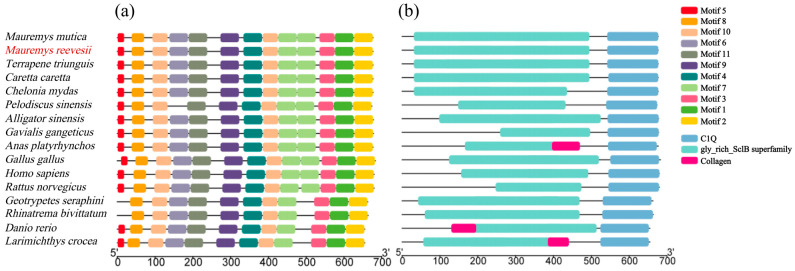
Conserved motifs and structural domain analysis of Col10a1 across different species. (**a**) Conserved motifs; (**b**) structural domain.

Through prediction of conserved amino acid regions, 11 highly conserved motifs were identified in Col10a1 proteins across diverse vertebrates (Figure 2a). Furthermore, Col10a1 proteins across these vertebrates contain identical structural domains, namely the gly_rich_SclB superfamily and C1q domain (Figure 2b). Among these, the gly_rich_SclB superfamily is most conserved in turtle species, while the C1q domain is highly conserved across various vertebrate lineages.

### 3.4. Expression Changes in Col10a1 in the Carapace of M. reevesii Embryos During Development

**Figure 3:**
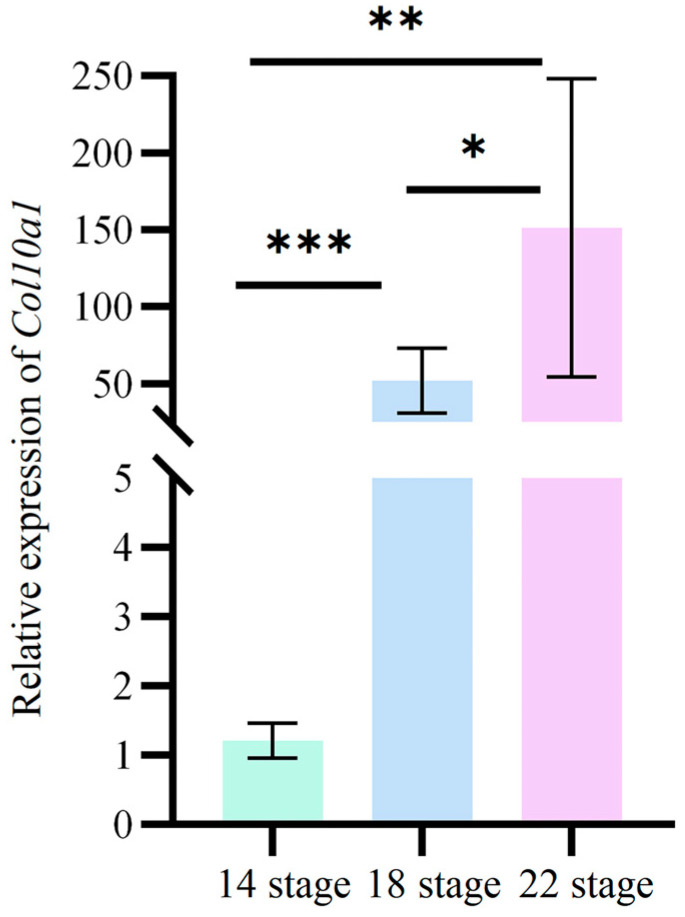
Relative expression of *Col10a1* in the carapace of *M. reevesii* embryos at developmental stages 14, 18, and 22. Significant differences are indicated by * (*p* < 0.05), ** (*p* < 0.01) and *** (*p* < 0.001). Gapdh was used as the internal reference gene for the 2^−ΔΔCt^ method. The original data are available in Supplementary Table S1.

RT-qPCR analysis was conducted to examine the expression dynamics of the *Col10a1* in carapace tissues of *M. reevesii* embryos at developmental stages 14, 18, and 22. The results (Figure 3) demonstrate that *Col10a1* expression increased gradually throughout embryonic development, reaching its highest level at stage 22. Expression at stage 22 is significantly higher than at stages 14 and 18.

### 3.5. Expression Profile of the Col10a1 in Adult M. reevesii Tissues

**Figure 4:**
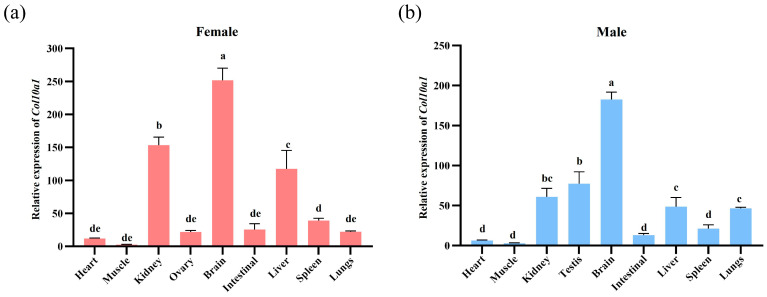
Relative expression of *Col10a1* in various tissues of three-winter-old *M. reevesii*. (**a**) expression of *Col10a1* in female individuals; (**b**) expression of *Col10a1* in male individuals. Different lowercase letters indicate significant differences between groups (*p* < 0.05). *Gapdh* was used as the internal reference gene for the 2^−ΔΔCt^ method. The original data are available in Supplementary Table S2.

The expression profile of the *Col10a1* in three-winter-old male and female *M. reevesii* tissues is shown below (Figure 4). The *Col10a1* was expressed in all examined tissues in both male and female adults, with highly similar expression patterns between sexes. Relatively high expression levels were observed in brain, kidney, and liver, whereas muscle showed the lowest expression. Among these, brain tissue demonstrates the most abundant expression, with significantly higher levels than other tissues (*p* < 0.05).

### 3.6. Effects of Salinomycin Sodium Salt and Oxymatrine on Gene Expression in MRCCs

**Figure 5:**
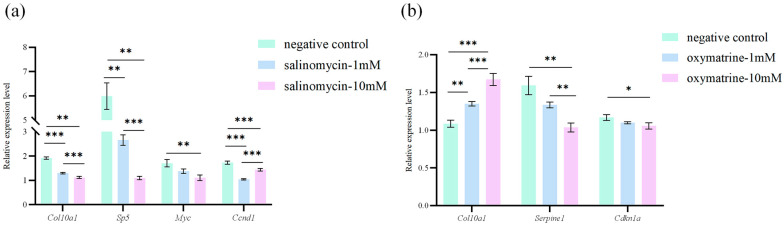
Relative expression of *Col10a1* in MRCCs after treatment with salinomycin sodium salt (**a**) and oxymatrine (**b**). Significant differences are indicated by * (*p* < 0.05), ** (*p* < 0.01) and *** (*p* < 0.001). *Gapdh* was used as the internal reference gene for the 2^−ΔΔCt^ method. The original data are available in Supplementary Table S3.

MRCCs were treated with different concentrations (1 mM and 10 mM) of salinomycin sodium salt and oxymatrine, as shown in Figure 5a,b, respectively. Compared to the negative control group (DMSO group), *Col10a1* expression in MRCCs decreased significantly in response to salinomycin sodium salt in a dose-dependent manner, with the 10 mM treatment group showing a highly significant reduction (*p* < 0.001). In contrast, both 1 mM and 10 mM doses of oxymatrine significantly upregulated *Col10a1* expression (*p* < 0.001). Furthermore, in MRCCs, treatment with salinomycin sodium salt downregulated the expression of Wnt/β-catenin signaling pathway target genes (*Sp5*, *Myc*, *Ccnd1*), whereas oxymatrine treatment reduced the expression of TGF-β/Smad signaling pathway target genes (*Serpine1*, *Cdkn1a*).

## 4. Discussion

This study successfully cloned the *Col10a1* gene from *M. reevesii*, which spans 2194 bp, including a 2034 bp coding sequence (CDS) that encodes a protein of 677 amino acids. The encoded Col10a1 protein is an alkaline-stable hydrophilic protein. Amino acid sequence alignment and ML phylogenetic tree analysis indicate that *M. reevesii* Col10a1 is most closely related to turtle and tortoise species within the reptilian clade. Protein structure determines function. The *M. reevesii* Col10a1 protein is predicted to be secreted and contains an N-terminal signal peptide that serves as a molecular localization signal. This guides the newly synthesized Col10a1 precursor protein into the endoplasmic reticulum (ER) for post-translational modification. Subsequently, the signal peptide is specifically cleaved by signal peptidase within the ER lumen, releasing the mature Col10a1 protein into the ECM [38]. The C-terminal NC1 domain of Col10a1 plays a pivotal role in collagen X assembly by driving the homotrimerization of three α1(X) chains, making it essential for Col10a1 secretion [39,40]. Furthermore, the NC1 domain contains a specific calcium ion-binding site; binding of calcium ions is known to promote the initiation of mineralization in growth plate cartilage [21].

RT-qPCR analysis revealed that *Col10a1* gene expression in the carapace of *M. reevesii* embryos exhibited distinct temporal characteristics, with expression levels progressively increasing throughout carapace development. Notably, *Col10a1* gene expression was significantly upregulated (*p* < 0.05) in carapace tissue during stage 22 of *M. reevesii* embryonic development. This expression pattern coincides with the biological processes of carapace ossification and collagen deposition during *M. reevesii* embryonic development. Previous studies indicate that during vertebrate skeletal development, the X-type collagen gene (*Col10a1*) is specifically expressed in the hypertrophic chondrocyte zone during endochondral ossification [19,41], and its upregulation is closely associated with cartilage matrix mineralization and the formation of ossification centers [42]. Furthermore, *Col10a1* facilitates ECM remodeling in the growth plate, providing a scaffold for vascular invasion and subsequent ossification, as well as guiding osteoblast migration and facilitating Type I collagen deposition on the mineralized cartilage surface [20,21]. Thus, our results suggest that during *M. reevesii* carapace development, elevated *Col10a1* expression may contribute to carapace bone plate formation by promoting collagen synthesis and mineralized matrix deposition.

Additionally, in studies of tissue expression profiles in adult *M. reevesii*, *Col10a1* was expressed in all tissues, with higher expression levels in the brain, kidneys, and liver. Its expression level in brain tissue was particularly significant (*p* < 0.05). *Col10a1* encodes the α1 chain of type X collagen, typically expressed in cartilage growth plates and involved in skeletal development [43]. However, its prominent expression in non-skeletal tissues such as the brain, kidney, and liver, however, suggests potential tissue-specific functions beyond osteogenesis. Sustained *Col10a1* expression in the adult brain may indicate a role in neural tissue homeostasis. Although the precise function of type X collagen in the brain remains incompletely understood, other collagen family members (such as type IV collagen) have been demonstrated to be critical for the nervous system. Studies have shown that type IV collagen is a core component of the blood–brain barrier basement membrane [44,45], which plays a key role in maintaining blood–brain barrier function [46]. Moreover, Type IV collagen plays a central role in arterial stiffness and dementia pathogenesis, with *Col4a1* mutations and dysregulation of its synthesis and degradation closely associated with vascular dysfunction and cognitive decline [47]. These findings suggest that *Col10a1* may participate in physiological or pathological processes of the nervous system by influencing brain ECM dynamics or vascular stability, though the precise mechanisms require further investigation. Furthermore, in the kidney, collagen serves as a critical structural component. Type IV collagen and type V collagen constitute the primary constituents of the glomerular basement membrane (GBM) and glomerular mesangial ECM, respectively [48,49], while the tubulointerstitial ECM contains types I, III, IV, V, VI, VII, and VIII collagen [49]. Recent studies reveal that type X collagen is upregulated during renal fibrosis, and *Col10a1* overexpression has been shown to promote aberrant ECM deposition following tubulointerstitial injury, thereby exacerbating renal fibrosis [50]. In the liver, *Col10a1* expression is associated with fibrosis regulation. It is induced during hepatic stellate cell activation, subsequently promoting collagen deposition and driving the progression of liver fibrosis [51].

Collagen metabolism is jointly regulated by a series of signaling pathways. Among these, the Wnt/β-catenin and TGF-β/Smad signaling pathways are classical pathways regulating collagen metabolism, primarily involved in the deposition of ECM [52,53,54,55]. Previous studies have established that salinomycin sodium salt is a potent Wnt/β-catenin pathway inhibitor, as it acts by blocking the phosphorylation of the Wnt coreceptor lipoprotein receptor related protein 6 (LRP6) and subsequently inducing its degradation [34,56,57]. In contrast, oxymatrine inhibits the TGF-β/Smad pathway through a dual mechanism, which involves suppressing the expression of Smad3 and its coactivator CBP while promoting the expression of the inhibitory Smad7 [35,58,59]. In this study, pathway inhibitor treatment of MRCCs revealed that salinomycin sodium salt downregulated *Col10a1* expression, whereas oxymatrine upregulated it. This opposing effect suggests that the signaling pathways targeted by the two drugs may have opposing regulatory effects on *Col10a1* expression. To further verify the pathway inhibition effects of salinomycin sodium salt and oxymatrine in MRCCs, we analyzed the expression of key target genes in the relevant pathways. *Sp5*, *Myc* and *Ccnd1* are established direct target genes of the Wnt/β-catenin pathway, such that pathway activation enables nuclear β-catenin to complex with TCF/LEF and initiate their transcription, whereas pathway inhibition impairs the formation or function of this complex and diminishes transcriptional output [60,61,62]. In contrast, *Serpine1* and *Cdkn1a* are classic direct transcriptional targets of the TGF-β/Smad signaling pathway, and as key markers of the pathway’s activity, their expression is precisely regulated through the interaction of Smad proteins with specific promoter elements [63,64]. The results of this study indicate that in MRCCs, treatment with salinomycin sodium salt significantly reduced the expression of Wnt/β-catenin target genes (*Sp5*, *Myc*, *Ccnd1*), while oxymatrine decreased that of TGF-β/Smad target genes (*Serpine1*, *Cdkn1a*), confirming that each drug effectively inhibited its respective target pathway. Therefore, it can be inferred that within MRCCs, salinomycin sodium salt and oxymatrine likely inhibit the Wnt/β-catenin and TGF-β/Smad pathways, respectively, mediating the suppression and enhancement of *Col10a1* expression, and revealing that the Wnt/β-catenin and TGF-β/Smad pathways function as positive and negative regulators of *Col10a1*, respectively. Evidence suggests that Wnt/β-catenin signaling promotes *Col10a1* expression by activating the transcription factor *Runx2*, mediating chondrocyte hypertrophy, accelerating chondrocyte maturation, and advancing endochondral ossification [65,66]. In contrast, within the TGF-β/Smad signaling pathway, TGF-β/Smads activate signaling that suppresses *Runx2* and *Col10a1* expression, reducing cartilage cell ECM degradation and thereby delaying cartilage cell hypertrophy [67,68,69]. Therefore, the deposition of collagen in *M. reevesii* carapace and the ossification process may be regulated by the antagonistic control of *Col10a1* expression through the Wnt/β-catenin and TGF-β/Smad signaling pathways. This precise regulatory mechanism ensures the timely deposition of collagen and orderly ossification within carapace tissue during the transition of chondrocytes toward hypertrophy.

However, this study has certain limitations. The precise mechanisms by which salinomycin and oxymatrine inhibit the Wnt/β-catenin and TGF-β/Smad pathways in MRCCs, respectively, are not fully elucidated, and their potential off-target effects remain to be evaluated. Consequently, elucidating their precise mechanisms of action will be an important future direction.

## 5. Conclusions

This study successfully cloned and characterized *Col10a1* from *M. reevesii*. Bioinformatics analysis revealed that *M. reevesii* Col10a1 is most closely related to other reptilian species. The *M. reevesii* Col10a1 is a basic, hydrophilic secretory molecule, whose secondary structure is dominated by α-helices and random coils and features a C-terminal NC1 domain. Expression analysis revealed *Col10a1* expression increases gradually during embryonic carapace development and is highly expressed in brain, kidneys, and liver of adult *M. reevesii*. Pharmacological inhibition experiments suggest that *Col10a1* expression is potentially positively regulated by the Wnt/β-catenin pathway and negatively regulated by the TGF-β/Smad pathway. These findings confirm the key role of *Col10a1* in collagen deposition and carapace ossification in *M. reevesii*, offer new insights into the underlying mechanisms, and lay a foundation for exploring Wnt/TGF-β crosstalk in carapace development and hardening.

## Figures and Tables

**Table 1:** Primers used for cDNA cloning and expression analysis of *M. reevesii*.

Name	Primer Sequences (5′—3′)	Purpose
*Col10a1*-F	CACTGCATGCCAGCGTATCT	cDNA cloning
*Col10a1*-R	ATCCTCACTTCCCCTTTGTGG	
*Col10a1*-qF	AAGCAATACTCCCACAGATGCCAGA	Real-time fluorescence quantification
*Col10a1*-qR	GGTAGGCTTTGGAGAGAATGGCAGAG	
*Sp5*-qF	CCCACCTTCCATGATCGCA	
*Sp5*-qR	TGGGTGTGCAGGAAAGGTC	
*Myc*-qF	GGGCCAAGATCTCACCCTTT	
*Myc*-qR	CTGGTGCCTGCTTTTGGAAG	
*Ccnd1*-qF	AAGGTCTACGGCAAGACCTCG	
*Ccnd1*-qR	TTGAGCTTCTTGTTCTGATGGGTC	
*Serpine1*-qF	ACGGGGAGCGCATATAAGG	
*Serpine1*-qR	CCACGTTACGATCTGGGGAC	
*Cdkn1a*-qF	ACGGGGAGCGCATATAAGG	
*Cdkn1a*-qR	CCACGTTACGATCTGGGGAC	
*Gapdh*-F	TGGGATACACCGAGGACC	Reference genes
*Gapdh*-R	CATACCAGGAGACCAGTTTGAC	

**Table 2:** NCBI accession numbers of the Col10a1 protein sequences across different species.

Species	NCBI Accession Number
*Mauremys mutica*	XP_044865908.1
*Caretta caretta*	XP_048700688.1
*Chelonia mydas*	XP_007057487.3
*Terrapene triunguis*	XP_026509269.1
*Pelodiscus sinensis*	XP_025040541.1
*Gavialis gangeticus*	XP_019375611.1
*Alligator sinensis*	XP_025062835.1
*Gallus gallus*	XP_046768794.1
*Anas platyrhynchos*	XP_071892193.1
*Homo sapiens*	NP_001411035.1
*Rattus norvegicus*	NP_037272.1
*Danio rerio*	NP_001077296.1
*Larimichthys crocea*	XP_010745936.1
*Rhinatrema bivittatum*	XP_029451094.1
*Geotrypetes seraphini*	XP_033795657.1

## Data Availability

The raw data analyzed in this study can be downloaded from the National Center for Biotechnology Information (NCBI) databases.

## Supplementary Materials

The following supporting information can be downloaded at: https://www.mdpi.com/article/10.3390/ani15223315/s1, Figure S1: Melting curve analysis of Col10a1 (a), Gadph (b), Sp5 (c), Myc (d), Ccnd1 (e), Serpine1 (f), Cdkn1a (g); Figure S2: Amplification efficiency analysis of *Gadph* (a), *Col10a1* (b), *Col10a1* (a), *Gadph* (b), *Sp5* (c), *Myc* (d), *Ccnd1* (e), *Serpine1* (f), *Cdkn1a* (g); Figure S3: Agarose gel electrophoresis image of PCR from Col10a1 gene cloned colonies; Figure S4: Alignment of the cloned *Col10a1* sequence from *M. reevesii* with the NCBI reference sequence (XM_039529706.1); Figure S5: (a) Nucleotide of *M. reevesii Col10a1* and its encoded protein sequence; (b) Prediction of hydrophilicity/hydrophobicity of *M. reevesii* Col10a1; (c) Phosphorylation prediction results of *M. reevesii* Col10a1; (d) Signal peptide prediction results of *M. reevesii* Col10a1; (e) *M. reevesii* Col10a1 secondary structure prediction; (f) *M. reevesii* Col10a1 tertiary structure prediction; (g) *M. reevesii* Col10a1 domains prediction; Table S1: Figure 3-Relative Expression Calculation Data for Col10a1; Table S2: Figure 4-Relative Expression Calculation Data for Col10a1; Table S3: Figure 5-Relative Expression Calculation Data for Col10a1.
