# Supplementary material for: Cloning and Expression of Col10a1 Gene and Its Response to Wnt/TGF-β Signaling Inhibitors in the Chinese Three-Keeled Pond Turtle (Mauremys reevesii)
Source: Animals (Basel). 2025 Nov 17;15(22):3315. doi: 10.3390/ani15223315 (PMC12649250; doi:10.3390/ani15223315)
Supplement: Supplementary file 1 [file animals-15-03315-s001.zip › Supplementary Figure .pdf]

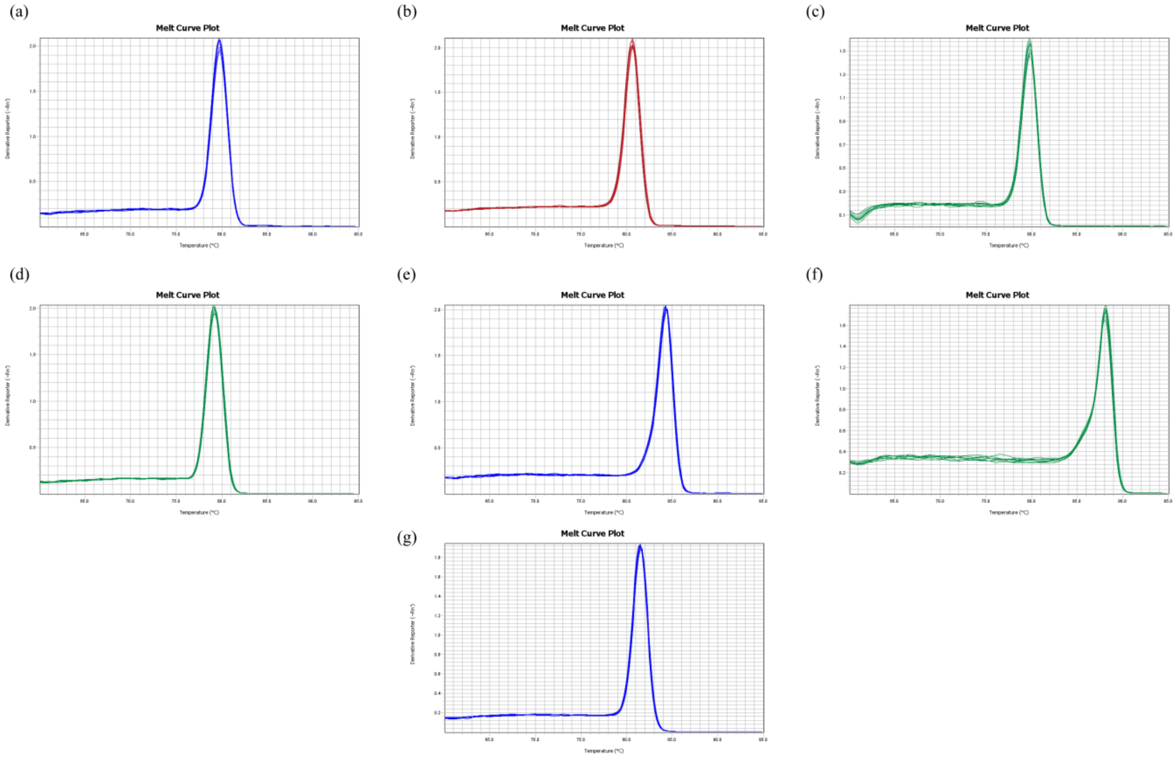

Figure S1. Melting curve analysis of *Col10a1* (a), *Gadph* (b), *Sp5* (c), *Myc* (d), *Ccnd1* (e), *Serpine1* (f), *Cdkn1a* (g).

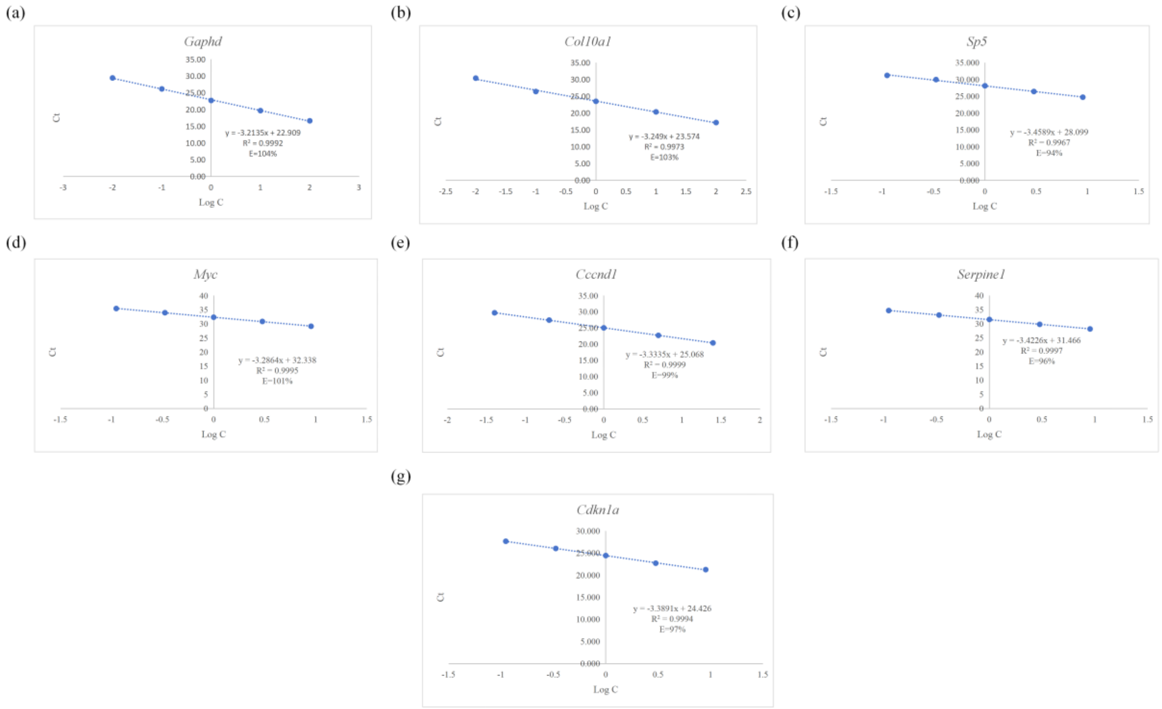

Figure S2. Amplification efficiency analysis of *Gadph* (a),*Col10a1* (b), *Col10a1* (a), *Gadph* (b), *Sp5* (c), *Myc* (d), *Ccnd1* (e), *Serpine1* (f), *Cdkn1a* (g).

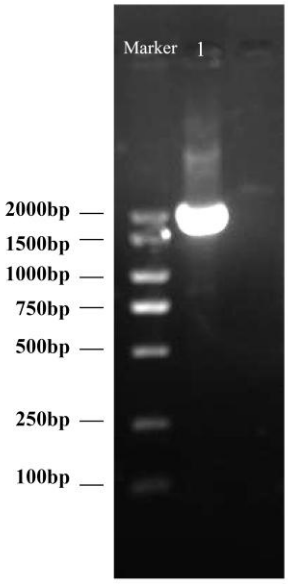

Figure S3. Agarose gel electrophoresis image of PCR from *Col10a1* gene cloned colonies.

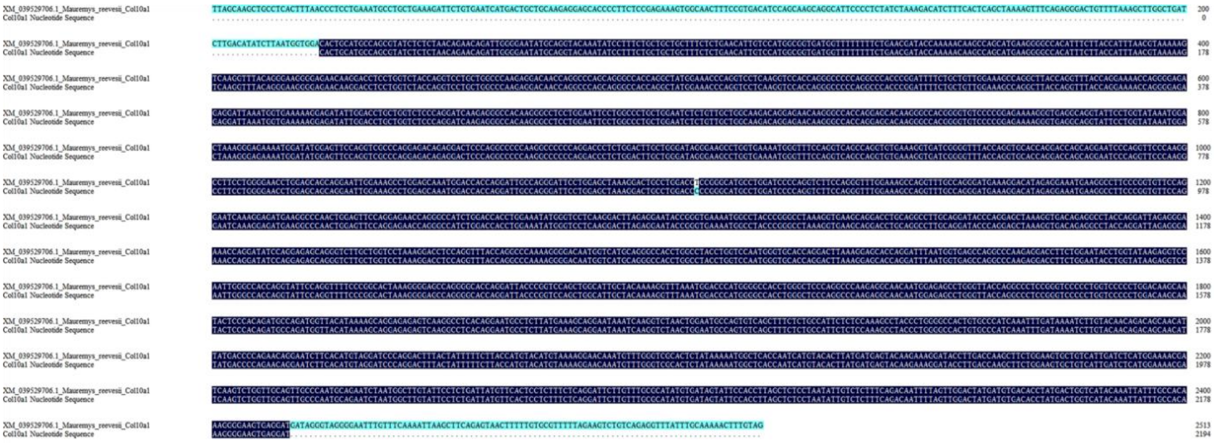

Figure S4. Alignment of the cloned *Col10a1* sequence from *M. reevesii* with the NCBI reference sequence (XM_039529706.1)

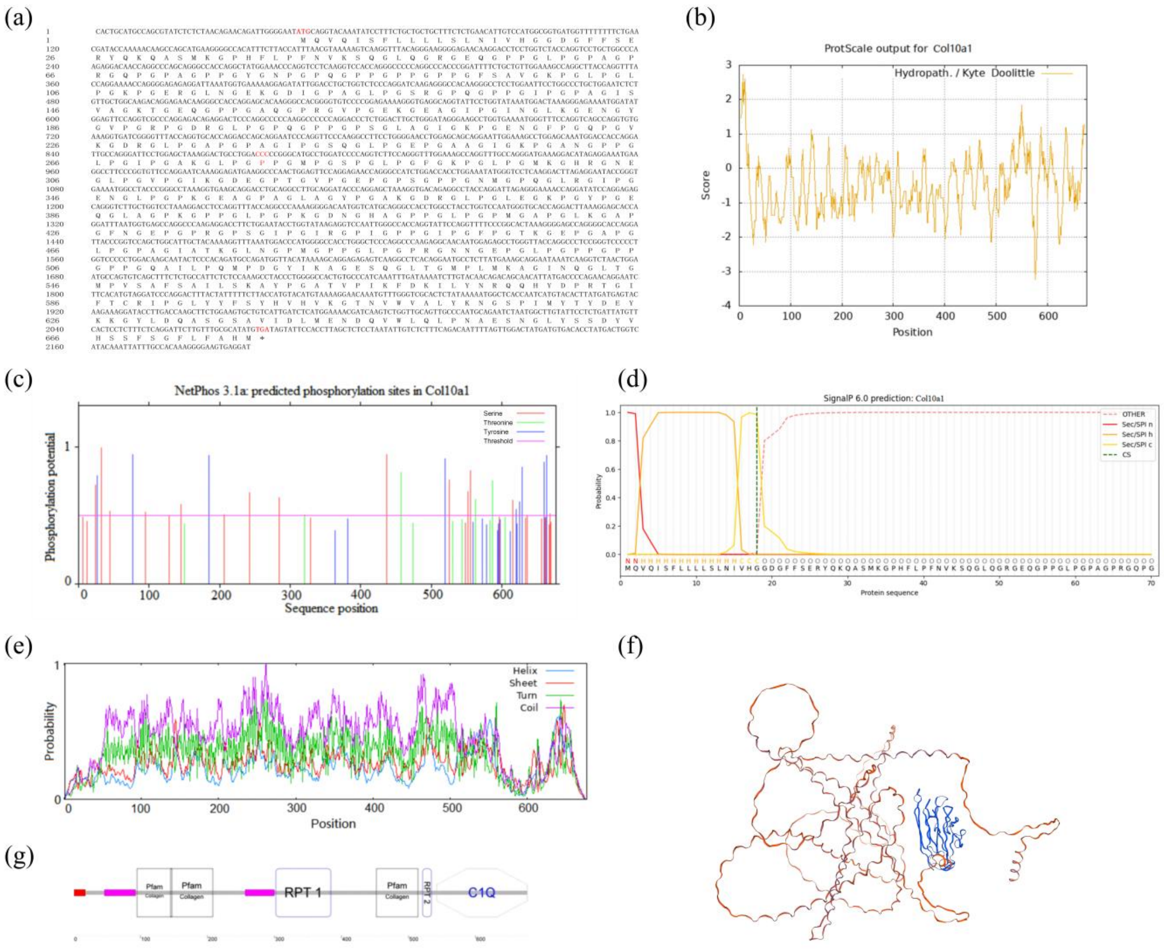

Figure S5. (a) Nucleotide of *M. reevesii Col10a1* and its encoded protein sequence; (b) Prediction of hydrophilicity/hydrophobicity of *M. reevesii* Col10a1; (c) Phosphorylation prediction results of *M. reevesii* Col10a1; (d) Signal peptide prediction results of *M. reevesii* Col10a1; (e) *M. reevesii* Col10a1 secondary structure prediction; (f) *M. reevesii* Col10a1 tertiary structure prediction; (g) *M. reevesii* Col10a1 domains prediction.
